# Beyond Chemoimmunotherapy: Emerging Cellular and Targeted Therapies in Transformed Follicular Lymphoma: A Scoping Review

**DOI:** 10.1177/10732748261454479

**Published:** 2026-06-09

**Authors:** Abdulrahman F. Al-Mashdali, Rola Ghasoub, Shrouq Hwafdeh, Mohamed A. Yassin

**Affiliations:** 1 Hematology and Bone Marrow Transplant Department, National Center for Cancer Care and Research, 36977 Hamad Medical Corporation, Doha, Qatar; 2 Pharmacy Department, National Center for Cancer Care and Research, 36977 Hamad Medical Corporation, Doha, Qatar

**Keywords:** transformed follicular lymphoma, histologic transformation, scoping review, CAR T-cell therapy, bispecific antibodies, selinexor

## Abstract

**Introduction:**

Transformed follicular lymphoma (t-FL) is an aggressive lymphoma with limited prospective evidence to guide treatment, particularly in the relapsed setting. We summarized the current evidence on emerging cellular and targeted therapies that extend beyond conventional chemoimmunotherapy.

**Methods:**

This scoping review was conducted in accordance with the PRISMA extension for Scoping Reviews (PRISMA-ScR) and structured using the Population–Concept–Context (PCC) framework. Adults with histologically confirmed or strongly suspected t-FL were the population of interest. Findings were synthesized narratively.

**Results:**

Seventeen studies met inclusion criteria across three therapeutic categories: CAR-T therapy (7 interventional trials, n = 130 t-FL patients in reported subsets), CD20×CD3 bispecific antibodies (BsAbs; n = 61 t-FL patients with extractable outcomes), and selinexor (n = 31 t-FL patients); together encompassing approximately 222 t-FL patients across primary interventional cohorts with extractable outcomes. CAR-T products achieved overall response rates (ORR) of 52–83% and complete response (CR) rates of 40–58%; randomized second-line trials favored axicabtagene ciloleucel and lisocabtagene maraleucel over standard care. In real-world CAR-T series, ORR ranged from 82–92% and CR rates from 64–67% across registry cohorts. BsAbs were active in heavily pretreated disease (epcoritamab ORR 50%/CR 44%; glofitamab ORR 55%/CR 35%), with predominantly low-grade cytokine release syndrome. Selinexor showed more modest efficacy (ORR 39%/CR 16%) but durable benefit in complete responders.

**Conclusions:**

Current evidence supports a stepwise treatment framework: DLBCL-like induction at transformation, autologous stem-cell transplant in fit, chemosensitive responders, CAR-T as the preferred option at first relapse, BsAbs after CAR-T or when cellular therapy is not feasible, and selinexor in later-lines of therapy. Additional prospective t-FL–inclusive trials are needed to refine treatment selection and biomarker-guided sequencing.

## Graphical abstract

**Graphical Abstract:**
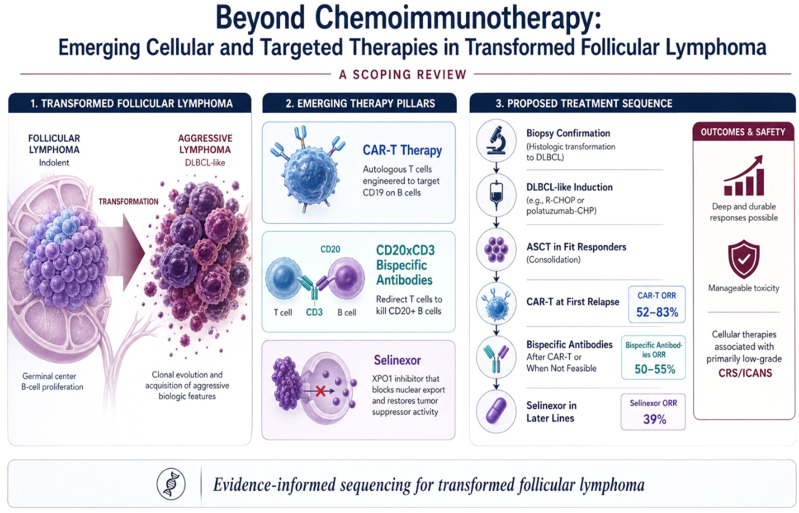

## Introduction

Follicular lymphoma (FL) is the second most common non-Hodgkin lymphoma and the most common indolent subtype.^1^ Although untreated patients typically survive for years, the natural history of FL can be dramatically altered by histologic transformation (HT) into a more aggressive lymphoma. The spectrum of indolent lymphomas susceptible to transformation includes FL, marginal zone lymphoma, lymphoplasmacytic lymphoma, and chronic lymphocytic leukemia/small lymphocytic lymphoma.^2^

Among these, FL transformation has been the most extensively characterized, occurring at a consistent rate of 1–2% per year.^2^ Transformation typically presents with rapid lymphadenopathy, extranodal disease, B symptoms, elevated lactate dehydrogenase (LDH), and occasional hypercalcemia.^2^ Although historical outcomes were poor, contemporary management has improved survival, particularly when transformation occurs in patients with limited prior therapy.^3-5^

Despite these advances, prospective evidence specifically addressing transformed disease remains scarce. Most current treatment standards are extrapolated from subset analyses of trials in aggressive lymphoma, and several pivotal registration programs explicitly excluded transformed histology. This scoping review summarizes the available evidence on the diagnosis and management of t-FL, with a focus on emerging cellular and targeted therapies, and proposes an evidence-informed management algorithm to support clinical decision-making and identify research priorities.

## Methods

### Protocol and Registration

This scoping review followed the Preferred Reporting Items for Systematic Reviews and Meta-Analyses extension for Scoping Reviews (PRISMA-ScR) checklist (Tricco et al, 2018), and the methodology of Arksey and O'Malley as refined by the Joanna Briggs Institute. The completed PRISMA-ScR checklist is provided as a supplementary file. A formal protocol was developed a priori and is available from the corresponding author on request. The review was not pre-registered; however, all eligibility criteria, search strategy elements, and data-charting variables were defined before screening.

## Review Questions and PCC Framework

The review was structured around the Population–Concept–Context (PCC) framework. The Population comprised adults (≥18 years) with FL and histologically confirmed or strongly suspected histologic transformation. The Concept covered diagnostic strategies and therapeutic approaches, including chemoimmunotherapy, autologous and allogeneic stem-cell transplantation, CD19-directed CAR-T therapy, CD20×CD3 bispecific antibodies, and novel targeted agents (e.g., selinexor), with reported efficacy and safety outcomes. The Context included all care settings (clinical-trial and real-world) and all geographic regions, incorporating peer-reviewed studies and major hematology meeting abstracts with extractable t-FL data.

### Eligibility Criteria

Eligible study designs were interventional trials and trial expansions (CAR-T, CD20×CD3 bispecific antibodies, selinexor); registries; and phase II/III randomized studies. Studies were required to report at least one efficacy endpoint — overall response rate (ORR), complete response (CR), duration of response (DOR), progression-free survival (PFS), event-free survival (EFS), or overall survival (OS) and/or safety outcomes including cytokine release syndrome (CRS), immune effector cell-associated neurotoxicity syndrome (ICANS), or grade ≥3 adverse events.

Reports published from 1 January 2000 through 31 May 2025, in any language, were considered. For mixed-histology datasets, inclusion required either explicit identification of a t-FL subset or sufficient detail to abstract t-FL–specific results. Single case reports, narrative editorials, and letters without primary data were excluded, as were studies with fewer than ten patients when no t-FL subgroup outcomes could be extracted, reports without separable t-FL data, pediatric-only studies, and purely diagnostic or radiologic reports.

### Information Sources and Search Strategy

A systematic search was performed in three electronic databases: PubMed/MEDLINE, EMBASE, and the Cochrane Library. This was supplemented by manual screening of conference proceedings from the American Society of Hematology (ASH) and the European Hematology Association (EHA) from 2020 to 2025, as well as hand-searching the reference lists of recent narrative reviews.

The search strategy combined controlled vocabulary terms, including MeSH and Emtree, with free-text terms grouped under three core concepts and linked using Boolean operators: (i) “transformed follicular lymphoma” OR “histologic transformation” OR “Richter-like transformation”; AND (ii) “CAR-T″ OR “chimeric antigen receptor” OR “axicabtagene” OR “lisocabtagene” OR “tisagenlecleucel” OR “bispecific antibody” OR “epcoritamab” OR “glofitamab” OR “mosunetuzumab” OR “selinexor” OR “XPO1” OR “autologous stem cell transplant”; AND (iii) “efficacy” OR “response” OR “survival” OR “safety”.

### Study Selection and Data Management

**Figure 1.**
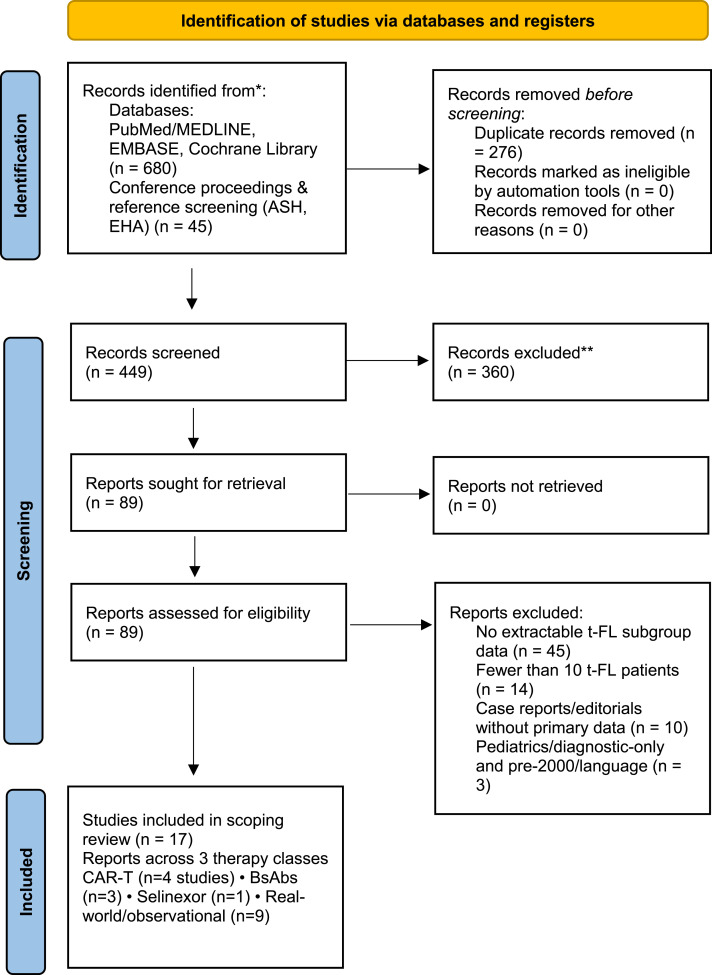
PRISMA-ScR flow diagram of the study selection process

All retrieved citations were imported into EndNote 21 and de-duplicated. Reasons for full-text exclusion were recorded and are summarized in the PRISMA-ScR flow diagram (Figure 1).

### Data Extraction

Extracted data items included bibliographic information, including first author, year, and country; study design and setting; sample size of patients with transformed follicular lymphoma; intervention and treatment line; key efficacy endpoints, including overall response rate (ORR), complete response (CR), duration of response (DOR), progression-free survival (PFS), event-free survival (EFS), and overall survival (OS); and safety outcomes, including cytokine release syndrome (CRS) grade, immune effector cell-associated neurotoxicity syndrome (ICANS), grade ≥3 adverse events, and treatment-related mortality.

### Evidence Synthesis and Risk-Of-Bias Assessment

Consistent with PRISMA-ScR guidance, no formal risk-of-bias assessment was performed, as the aim was to map the breadth and nature of available evidence rather than to generate pooled comparative estimates. Quantitative meta-analysis was not performed because of marked clinical and methodological heterogeneity. Limitations of the underlying evidence base, including selection bias, small t-FL subgroups, and heterogeneous transformation definitions, are discussed in the limitations section.

## Results

### Selection of Sources of Evidence

The database search retrieved 680 records, supplemented by 45 records from conference proceedings and reference screening. After removal of 276 duplicates, 449 records were screened by title and abstract; 89 full texts were assessed for eligibility, and 17 studies fulfilled the inclusion criteria (Figure 1). The most frequent reason for full-text exclusion was lack of extractable t-FL data (n = 45).

### Characteristics of Included Studies

**Figure 2.**
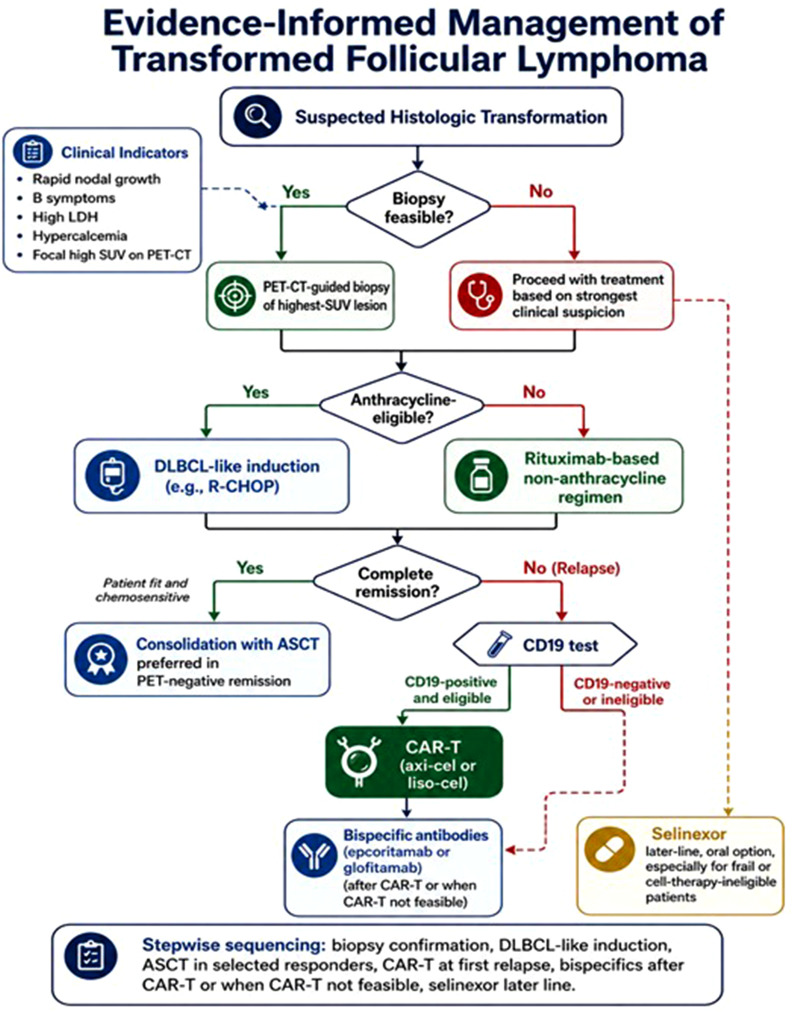
Evidence-informed management algorithm for transformed follicular lymphoma. ASCT, autologous stem-cell transplant; BR, bendamustine-rituximab; CAR-T, chimeric antigen receptor T-cell therapy; CR, complete response; ORR, overall response rate; POD24, progression of disease within 24 months

**Figure 3.**
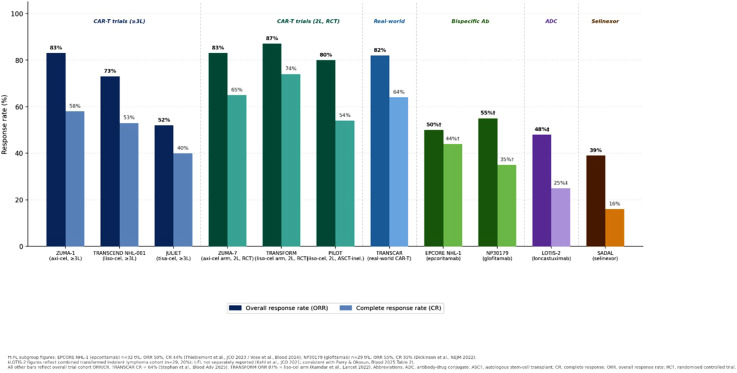
Overall response rate (ORR) and complete response (CR) rate across included studies in transformed follicular lymphoma, stratified by therapeutic class

**Figure 4.**
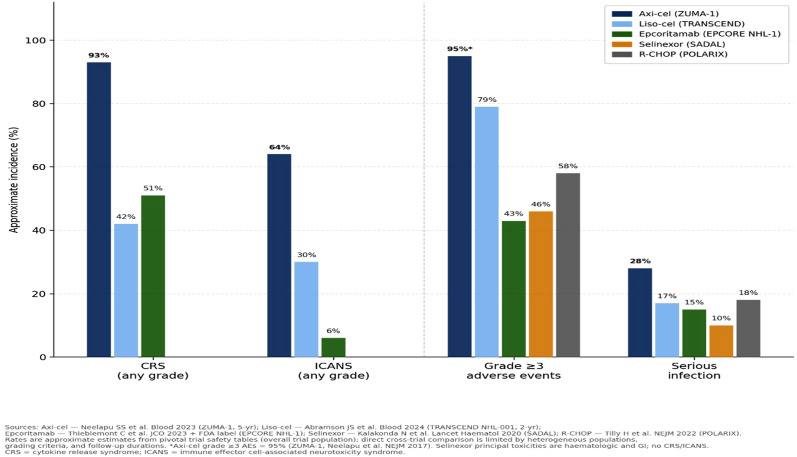
Key safety signals across therapy classes in transformed follicular lymphoma. Rates are approximate estimates from pivotal trial safety tables and represent the overall trial population (t-FL subgroups were too small for separate safety reporting in most trials). CRS = cytokine release syndrome; ICANS = immune effector cell-associated neurotoxicity syndrome

Of the 17 included studies, the primary interventional cohorts reported extractable t-FL data across three therapeutic categories: CAR-T (7 trials; n = 130 t-FL patients in reported subsets), CD20×CD3 bispecific antibodies (3 studies; n = 61 t-FL patients with extractable outcomes), and selinexor (1 study; n = 31 t-FL patients), totaling approximately 222 t-FL patients with extractable outcomes. t-FL proportions across the CAR-T trials ranged from 5–22% per treatment arm. The remaining studies provided observational, real-world, or supportive evidence informing the role of chemoimmunotherapy and ASCT. Synthesized findings are summarized in evidence tables (Tables 1-5), in a practical-positioning matrix (Table 6), and in the proposed management algorithm (Figure 2). The ORR and CR rates across included studies, stratified by therapeutic class, are summarized in Figure 3, while key safety signals across treatment modalities are shown in Figure 4.

**Table 1.** Observational and Chemoimmunotherapy Studies in Transformed Follicular Lymphoma

Study	Population	Treatment approach	Key outcomes	Notes
Retrospective cohort^6^	60 biopsy-proven t-FL	R-CHOP in 59% (others varied)	Median OS 50 mo; 5-yr OS 66%	DLBCL-type salvage patterns
National LymphoCare Study (NLCS)^7^	379 t-FL (147 biopsy; 232 clinical)	Rituximab mono 26%; rituximab-based CIT 35%	Biopsy-proven: median PFS 12 mo; Overall OS 60 mo	Prospective registry
PRIMA post-hoc analysis^8^	Treatment-naïve FL who later transformed	DLBCL-type salvage chemoimmunotherapy	5-yr OS post-transformation ∼48–50%	Post-hoc; first-line CIT responders

DLBCL, diffuse large B-cell lymphoma; OS, overall survival; PFS, progression-free survival; t-FL, transformed follicular lymphoma; R-CHOP, rituximab-cyclophosphamide-doxorubicin-vincristine-prednisone.

**Table 2.** CD19-Directed CAR-T Interventional Trials in Transformed Follicular Lymphoma

Study	Setting/Line	t-FL cases	Intervention	Efficacy	Key outcomes/Notes
ZUMA-1^9^	R/R LBCL incl. t-FL (≥3L)	n = 16 (67% of tFL cohort 2)	Axicabtagene ciloleucel (axi-cel)	ORR 83%, CR 58%; median PFS 5.9 mo; median OS 25.8 mo	Durable responses; CD19-neg. relapses reported
TRANSCEND NHL-001^10^	R/R LBCL incl. t-FL (≥3L)	n = 60 (22%; N = 270)	Lisocabtagene maraleucel (liso-cel)	ORR 73%, CR 53%; median PFS 6.8 mo; median OS 27.3 mo	Active in high-risk subtypes
PILOT^11^	ASCT-ineligible 2L DLBCL	n = 9 (15%; N = 61 infused)	Lisocabtagene maraleucel	ORR 80%, CR 54%; median PFS 9.0 mo; median OS NR	Durable CRs in frail/ASCT-ineligible
JULIET^12^	R/R DLBCL incl. t-FL (≥3L)	n = 21 (18%; N = 115 infused)	Tisagenlecleucel (tisa-cel)	ORR 52%, CR 40%; median PFS 2.9 mo; median OS 11.1 mo	Lower durability vs other CAR-T products
ZUMA-7^13^	2L randomized vs SOC	n = 19 (11%) axi-cel arm; n = 27 (15%) SOC arm	Axicabtagene ciloleucel	CR 65% vs 32%; EFS 8.3 vs 2.0 mo; PFS 14.7 vs 3.7 mo; OS NR vs 31 mo (axi-cel vs SOC)	EFS 8.3 vs 2.0 mo; positive RCT
TRANSFORM^14^	2L randomized vs SOC	n = 5 (5.4%) liso-cel arm; n = 6 (6.5%) SOC arm	Lisocabtagene maraleucel	CR 74% vs 43%; PFS NR vs 6.2 mo; OS NR vs 29.9 mo (liso-cel vs SOC)	Positive randomized 2L trial
BELINDA^15^	2L randomized vs SOC	Included; t-FL not separately reported in primary publication	Tisagenlecleucel	No superiority vs SOC	Negative 2L RCT

ASCT, autologous stem-cell transplant; CR, complete response; DLBCL, diffuse large B-cell lymphoma; EFS, event-free survival; LBCL, large B-cell lymphoma; ORR, overall response rate; PFS, progression-free survival; PMBCL, primary mediastinal B-cell lymphoma; R/R, relapsed/refractory; SOC, standard of care; t-FL, transformed follicular lymphoma; ≥3L, third line or later.

**Table 3.** CD20×CD3 Bispecific Antibody Trials in Transformed Follicular Lymphoma

Study	Agent	Setting	t-FL Cases	Efficacy (t-FL)	Durability/Safety
EPCORE NHL-1^16^	Epcoritamab	R/R LBCL; post-CAR-T allowed	n = 32	ORR 50%, CR 44%	Median DOR NR; 36-mo response 55%; CRS grade 1–2 predominant; ICANS ∼6%
NP30179 (Phase I/II)^17^	Glofitamab	R/R LBCL	n = 29	ORR 55.2%, CR 34.5%	Median DOR 5.5 mo; PFS plateau ∼24% at 8 mo; low-grade CRS common
STARGLO/OLYMPIA-5^18,19^	Odronextamab/Glofitamab	Pivotal programs	Excluded	—	Exclusion limits generalizability to t-FL

CAR-T, chimeric antigen receptor T-cell therapy; CR, complete response; CRS, cytokine release syndrome; DOR, duration of response; FL, follicular lymphoma; ICANS, immune effector cell-associated neurotoxicity syndrome; LBCL, large B-cell lymphoma; NR, not reached; ORR, overall response rate; PFS, progression-free survival; R/R, relapsed/refractory; t-FL, transformed follicular lymphoma.

**Table 4.** Selinexor as a Targeted Agent in Transformed Follicular Lymphoma

Study	Agent	Population	Efficacy (t-FL Subgroup)	Regulatory/Guidance
SADAL (Phase 2b)^20^	Selinexor (XPO1 inhibitor)	R/R DLBCL incl. Transformed indolent; t-FL n = 31 (∼24%)	ORR 38.7%, CR 16.1%; Median DOR 23.2 mo (CRs); Median OS 9.1 mo overall	FDA accelerated approval 2020; NCCN 2025 Cat 2A (t-FL after ≥2 lines)

CR, complete response; DLBCL, diffuse large B-cell lymphoma; DOR, duration of response; FDA, U.S. Food and Drug Administration; NCCN, National Comprehensive Cancer Network; NR, not reached; ORR, overall response rate; OS, overall survival; R/R, relapsed/refractory; t-FL, transformed follicular lymphoma; XPO1, exportin-1.

**Table 5.** Real-World, Case-Based, and Preclinical Evidence in Transformed Follicular Lymphoma

Study	Population	Intervention	Outcomes	Notes
Real-world CD19 CAR-T (China)^21^	14 t-FL incl. blastoid variant	CD19 CAR-T	ORR 92%; 2-yr PFS 67%, OS 73%	Small single-center series
US registry/ASH abstract^22^	t-FL vs de novo DLBCL (real-world)	CD19 CAR-T	Improved OS/PFS vs historical	Abstract-level evidence
Case: CAR-T + ASCT^23^	TP53-mut. bulky CD20-neg. t-FL	CD19 CAR-T then ASCT	Complete metabolic remission at 1 mo	Single case
Case: CD20/CD30 Bispecific CAR-T^24^	Bulky CD19-neg. t-FL	CD20/CD30 bispecific CAR-T	12+ mo remission; no CRS/ICANS	Single case
Preclinical: CD19/CD22 dual CAR-T^25^	FL/t-FL models (CD19-loss)	CD19/CD22 dual CAR-T	Preclinical efficacy; escape mitigation	Laboratory study
LOTIS-2^26^	n = 29 (20%) transformed indolent lymphoma; t-FL not separately reported	Loncastuximab tesirine (ADC)	Overall cohort: ORR 48%, CR 25%; PFS 4.9 mo; OS 9.5 mo (t-FL not separately analyzed)	ADC class; Parry & Okosun (Blood 2025) Table 2; t-FL not separately analyzed

ASCT, autologous stem-cell transplant; CAR-T, chimeric antigen receptor T-cell therapy; CRS, cytokine release syndrome; DLBCL, diffuse large B-cell lymphoma; FL, follicular lymphoma; ICANS, immune effector cell-associated neurotoxicity syndrome; ORR, overall response rate; OS, overall survival; PFS, progression-free survival; t-FL, transformed follicular lymphoma.

**Table 6.** Practical Positioning of Available Therapies in Transformed Follicular Lymphoma

Therapy/Approach	Typical Clinical Setting	Main Strengths	Main Limitations	Practical Role in Current Care
R-CHOP–based chemoimmunotherapy	Newly transformed, anthracycline-naïve t-FL	Widely available; familiar DLBCL backbone; supported by historical cohorts	Suboptimal in high-risk disease; limited prospective t-FL data	Standard first-line approach for most newly transformed cases
Dose-adjusted R-EPOCH	High-grade/double-hit biology (MYC+BCL2)	More intensive option for adverse biology	Greater toxicity; t-FL evidence remains extrapolative	Consider in selected high-grade/double-hit cases
Autologous stem-cell transplant (ASCT)	Fit, chemosensitive patients; PET-negative remission	Can prolong remission; durable disease control in selected patients	Retrospective evidence; uncertain OS advantage; transplant toxicity	Consolidation in selected PET-responsive, fit patients
CD19-directed CAR-T therapy	R/R t-FL; first relapse in eligible patients	Strongest evidence base; deep, durable remissions; FDA-approved	Access; manufacturing time; CRS/ICANS; CD19-neg. relapse	Preferred option at first relapse for eligible patients
CD20×CD3 bispecific antibodies	Post-CAR-T relapse or CAR-T-ineligible patients	Off-the-shelf access; meaningful activity; manageable toxicity	Limited t-FL-specific data; pivotal trials often excluded t-FL	Key later-line option; alternative when CAR-T not feasible
Selinexor	Later-line R/R disease; frail/non-cellular therapy candidates	Oral; distinct mechanism; FDA-labelled for t-FL	More modest efficacy; nausea, fatigue, thrombocytopenia	Later-line option when cellular/bispecific therapy unsuitable

ASCT, autologous stem-cell transplant; BR, bendamustine-rituximab; CAR-T, chimeric antigen receptor T-cell therapy; CRS, cytokine release syndrome; DLBCL, diffuse large B-cell lymphoma; ICANS, immune effector cell-associated neurotoxicity syndrome; OS, overall survival; R-CHOP, rituximab-cyclophosphamide-doxorubicin-vincristine-prednisone; R-EPOCH, rituximab-etoposide-prednisone-vincristine-cyclophosphamide-doxorubicin; t-FL, transformed follicular lymphoma.

Notably, five pivotal registration programs (ZUMA-5, ELARA, TRANSCEND FL, STARGLO, OLYMPIA-5) excluded transformed histology, despite broader approvals in DLBCL settings.^18,27-29^ This persistent exclusion underscores the need for dedicated, t-FL–specific prospective studies to refine treatment selection and sequencing.

### CAR-T Therapy in t-FL

Seven interventional CAR-T trials with extractable t-FL subgroup data (total n = 130 t-FL patients across reported subsets; Parry and Okosun, Blood 2025) informed the CAR-T evidence base (Table 2). In TRANSCEND NHL-001, 60 patients with t-FL (22% of 270 treated) received lisocabtagene maraleucel and achieved ORR 73%/CR 53% with a median PFS of 6.8 months.^10^ ZUMA-1 included a t-FL subset treated with axicabtagene ciloleucel (ORR 83%/CR 58%, with sustained CR rates).^9^ JULIET (tisagenlecleucel) showed lower durability in t-FL relative to other products.^12^ Among randomized second-line trials, ZUMA-7 (axi-cel) and TRANSFORM (liso-cel) demonstrated superiority over standard chemo-transplant strategies (CR 65% vs 32% and 74% vs 43%, respectively), whereas BELINDA (tisa-cel) did not.^13-15^ Real-world data corroborate meaningful activity. In the TRANSCAR registry, 60 patients with transformed indolent NHL treated with CAR-T achieved ORR 82% and CR 64%, with superior outcomes compared to de novo LBCL.^22^ A complementary US multicenter analysis of 691 patients (139 t-FL, 20%) similarly demonstrated improved PFS and OS for t-FL versus de novo DLBCL following CAR-T therapy.^22^

## CD20×CD3 bispecific Antibodies

Two BsAb studies provided extractable t-FL outcomes (epcoritamab and glofitamab; n = 61 t-FL patients). A third study of mosunetuzumab enrolled 23 t-FL patients within an 88-patient DLBCL/t-FL cohort, but did not report t-FL outcomes separately, precluding efficacy extraction.^26,30^ BsAb activity in heavily pretreated disease (Table 3). In EPCORE NHL-1, 32 patients with t-FL achieved ORR 50%/CR 44%, with 55% responses ongoing at 36 months.^16^ NP30179 (glofitamab) reported ORR 55.2%/CR 34.5% with median DOR of 5.5 months in 29 t-FL patients.^17^ Cytokine release syndrome was predominantly low grade and manageable with step-up dosing and corticosteroid prophylaxis.

### Selinexor

Selinexor remains the only non-cellular agent with FDA labeling that explicitly includes t-FL (Table 4). The pivotal SADAL phase 2b study enrolled 31 patients with t-FL (24% of 134) and reported ORR 38.7%/CR 16.1%, numerically higher than the de novo DLBCL subgroup (ORR 26.2%).^20^ Among complete responders, the median DOR was 23.2 months, and median OS in the t-FL subgroup was 9.1 months.^31^

### Real-World, Case-Based, and Preclinical Evidence

Six additional reports provided supportive evidence, including real-world CAR-T cohorts, an antibody-drug conjugate study with a t-FL subgroup (LOTIS-2), single-patient experiences with bispecific or sequential CAR-T strategies, and a preclinical CD19/CD22 dual-targeting program addressing CD19-loss escape (Table 5).

## Summary of Findings

### Biology and Pathogenesis

Transformation of FL is associated with retained lineage but higher-grade biology. Most cases remain germinal-center B-cell type, with a smaller subset shifting to activated B-cell phenotype. MYC deregulation is a key event and, when combined with BCL2 rearrangement, defines double-hit biology associated with inferior outcomes.^32,33^ Cooperating lesions in cell-cycle regulation, DNA-damage response, immune escape, and survival signaling are common. ABC patterns frequently involve MYD88, CD79B, and BCL10, whereas GCB patterns more often show REL amplification and TP53/CDKN2A/B abnormalities.^32,34-36^ Multi-omic studies confirm clonal relatedness between t-FL and antecedent FL through shared t(14;18) breakpoints and aberrant somatic hypermutation patterns.^32,37^ Whole-genome analyses describe biologically distinct FL subtypes, including DLBCL-like FL with greater transformation risk, and microenvironmental remodeling marked by reduced T follicular helper cells and increased exhausted T-cell populations.^38-46^ Collectively, these data support PET-directed re-biopsy of the highest-SUV lesion and molecular evaluation at transformation, including MYC/BCL2/BCL6 testing.^47^

### Epidemiology and Clinical Presentation

Approximately 10–15% of patients with FL experience HT during the disease course, at an annual rate of 1–2% across multiple cohorts, though contemporary rituximab-era registries suggest a lower cumulative rate of 4–13% at 10 years.^6,7,48^ Two large cohorts illustrate this risk and the value of biopsy^49^: a prospective study of >600 newly diagnosed FL patients reported HT in 11% over a median of 5 years, with biopsy confirmation in 85%^6^; in the PRIMA trial, 21% of 194 patients with biopsy-proven relapse showed transformation, and transformation was associated with markedly shorter time to recurrence (9.6 vs 22.8 months).^8^ Established baseline risk factors include multiple extranodal sites, ECOG performance status >1, elevated LDH, B symptoms, advanced stage, low albumin, high FLIPI/IPI, failure to achieve CR after first-line therapy, and POD24.^4^ Treatment exposure may also influence transformation risk, with population-based data suggesting higher t-FL rates after rituximab–bendamustine than after R-CVP.^42^ Early transformation (<18 months) is associated with substantially worse 5-year survival than later events.^6,50^ Clinically, HT should be suspected with rapid nodal growth, focal SUV >10, unexpected extranodal involvement, early treatment failure, B symptoms, or hypercalcemia; biopsy remains the diagnostic gold standard despite documented underuse.^42,51-53^

### Current and Emerging Treatment Strategies

Treatment of t-FL at transformation remains anchored in DLBCL-directed chemoimmunotherapy. R-CHOP is the standard for most anthracycline-naïve patients,^7,8,48^ whereas dose-adjusted R-EPOCH is reserved for high-grade biology with MYC and BCL2 rearrangements.^54-56^ Non-anthracycline rituximab-based regimens (BR or polatuzumab-based combinations) are options when anthracyclines are contraindicated.^57^ ASCT remains a selective option in fit, chemosensitive responders, with the strongest signal for durable disease control in PET-negative remission^58-61^; randomized t-FL–specific evidence is lacking, and treatment-related morbidity must be balanced against potential benefit (Table 1).

Among emerging therapies, CD19-directed CAR-T currently has the strongest evidence base in relapsed/refractory t-FL (Table 2) and is the preferred option at first relapse for eligible patients. Confirmation of CD19 expression is essential, and CD19-negative relapse is an established mechanism of post-CAR-T resistance. 11^10^, ^12^ and 62 CD20×CD3 bispecific antibodies (Table 3) provide an off-the-shelf alternative after CAR-T failure or when CAR-T is not feasible because of age, comorbidity, logistics, or access.^17,26,30,63^ Their toxicity profile is generally manageable and is dominated by low-grade CRS.^17,63^ Selinexor (Table 4) remains a useful later-line option, particularly for frail or heavily pretreated patients, and is the principal targeted agent with regulatory inclusion of t-FL.^20,31^

### Practical Positioning and Proposed Algorithm

The practical positioning of available modalities is summarized in Table 6, and an evidence-informed management algorithm is presented in Figure 2. In brief, transformation should be biopsy-confirmed whenever feasible (PET-CT-guided when possible)^64,65^; first-line treatment follows a DLBCL-like strategy tailored to disease biology and prior anthracycline exposure; ASCT is offered to fit, chemosensitive responders^61^; CAR-T is prioritized at first relapse in eligible patients; and bispecific antibodies and selinexor are reserved for later lines or CAR-T-ineligible settings.

## Limitations

This scoping review is descriptive by design and was intended to map the available evidence rather than generate pooled comparative estimates; accordingly, no formal risk-of-bias assessment or meta-analysis was performed, consistent with PRISMA-ScR guidance. Several limitations in the underlying literature should be acknowledged. First, many pivotal trials excluded transformed histology or reported only small t-FL subgroups, creating underrepresentation and potential selection bias. Second, definitions of transformation and diagnostic methods varied across studies, including biopsy-proven and clinically suspected cases.^5,7^ Third, heterogeneity in prior therapy, anthracycline exposure, timing of transformation, and biomarker characterization (e.g., double-hit status, TP53 alterations, cell of origin) limits external validity. Fourth, real-world datasets often lack centralized pathology review, standardized response assessment, and complete follow-up, with possible overlap between cohorts. Finally, several findings derived from subgroup analyses, post-hoc reports, or conference abstracts with immature follow-up, increasing the risk of selective reporting. Few studies directly address optimal sequencing, including CAR-T versus early bispecific use, or the management of CD19-negative relapse.

## Conclusion

Although the evidence base for transformed follicular lymphoma remains limited, the current literature supports a coherent treatment framework. Biopsy confirmation should be pursued whenever feasible, and initial management should follow a DLBCL-like approach tailored to disease biology and patient fitness. ASCT remains an option for selected chemosensitive patients, while CAR-T is the preferred strategy at first relapse in eligible patients. Bispecific antibodies provide an important option after CAR-T failure or when cellular therapy is not feasible, and selinexor is best reserved for later-line or oral-therapy settings. Across these therapies, careful toxicity management and infection prophylaxis remain essential. Future progress will depend on prospective, t-FL–inclusive trials and biomarker-guided sequencing studies.

## Supplemental Material

Supplemental Material - Beyond Chemoimmunotherapy: Emerging Cellular and Targeted Therapies in Transformed Follicular Lymphoma: A Scoping Review Supplemental Material for Beyond Chemoimmunotherapy: Emerging Cellular and Targeted Therapies in Transformed Follicular Lymphoma: A Scoping Review by Abdulrahman F. Al-Mashdali, Rola Ghasoub, Shrouq Hwafdeh, and Mohamed A. Yassin in Cancer Control.

## Data Availability

No new datasets were generated or analyzed in this study. All data supporting the findings of this review are available within the article and the cited references.*

## References

[1] Alaggio R Amador C Anagnostopoulos I , et al. The 5th edition of the World Health Organization Classification of Haematolymphoid Tumours: Lymphoid Neoplasms. Leukemia. 2022;36:1720-1748. 35732829 10.1038/s41375-022-01620-2 PMC9214472

[2] Smith S . Transformed lymphoma: what should I do now? Hematology Am Soc Hematol Educ Program. 2020;2020:306-311. 33275671 10.1182/hematology.2020000115 PMC7727564

[3] Alderuccio JP Lossos IS Rodrigues E , et al. Utilizing clinical transformation criteria for prognostic stratification in follicular lymphoma prior to initial immunochemotherapy. Hematol Rep. 2024;16(4):757-768. 10.3390/hematolrep16040060 PMC11503408 39449303

[4] Zheng W Liu M Guan L Wang S . Outcomes of the transformation of follicular lymphoma to diffuse large B-cell lymphoma in the rituximab era: a population-based study. Cancer Med. 2024;13:e7120. 38629251 10.1002/cam4.7120 PMC11022146

[5] Florindez JA Chihara D Reis IM Lossos IS Alderuccio JP . Risk of transformation by frontline management in follicular and marginal zone lymphomas: a US population-based analysis. Blood Adv. 2024;8:4423-4432. 38954843 10.1182/bloodadvances.2024013499 PMC11375286

[6] Link BK Maurer MJ Nowakowski GS , et al. Rates and outcomes of follicular lymphoma transformation in the immunochemotherapy era: a report from the University of Iowa/Mayo Clinic SPORE Molecular Epidemiology Resource. J Clin Oncol. 2013;31:3272-3278. 23897955 10.1200/JCO.2012.48.3990 PMC3757293

[7] Wagner-Johnston ND Link BK Byrtek M , et al. Outcomes of transformed follicular lymphoma in the modern era: a report from the National LymphoCare Study (NLCS). Blood. 2015;126:851-857. 26105149 10.1182/blood-2015-01-621375 PMC4543911

[8] Sarkozy C Trneny M Xerri L , et al. Risk factors and outcomes for patients with follicular lymphoma who had histologic transformation after response to first-line immunochemotherapy in the PRIMA trial. J Clin Oncol. 2016;34:2575-2582. 27298402 10.1200/JCO.2015.65.7163

[9] Neelapu SS Jacobson CA Ghobadi A , et al. Five-year follow-up of ZUMA-1 supports the curative potential of axicabtagene ciloleucel in refractory large B-cell lymphoma. Blood. 2023;141:2307-2315. 36821768 10.1182/blood.2022018893 PMC10646788

[10] Abramson JS Palomba ML Gordon LI , et al. Two-year follow-up of lisocabtagene maraleucel in relapsed or refractory large B-cell lymphoma in TRANSCEND NHL 001. Blood. 2024;143:404-416. 37890149 10.1182/blood.2023020854

[11] Sehgal A Hoda D Riedell PA , et al. Lisocabtagene maraleucel as second-line therapy in adults with relapsed or refractory large B-cell lymphoma who were not intended for haematopoietic stem cell transplantation (PILOT): an open-label, phase 2 study. Lancet Oncol. 2022;23:1066-1077. 35839786 10.1016/S1470-2045(22)00339-4

[12] Schuster SJ Bishop MR Tam CS , et al. Tisagenlecleucel in adult relapsed or refractory diffuse large B-cell lymphoma. N Engl J Med. 2019;380:45-56. 30501490 10.1056/NEJMoa1804980

[13] Locke FL Miklos DB Jacobson CA , et al. Axicabtagene ciloleucel as second-line therapy for large B-cell lymphoma. N Engl J Med. 2022;386:640-654. 34891224 10.1056/NEJMoa2116133

[14] Kamdar M Solomon SR Arnason J , et al. Lisocabtagene maraleucel versus standard of care with salvage chemotherapy followed by autologous stem cell transplantation as second-line treatment in patients with relapsed or refractory large B-cell lymphoma (TRANSFORM): results from an interim analysis of an open-label, randomised, phase 3 trial. Lancet. 2022;399:2294-2308. 35717989 10.1016/S0140-6736(22)00662-6

[15] Bishop MR Dickinson M Purtill D , et al. Second-line tisagenlecleucel or standard care in aggressive B-cell lymphoma. N Engl J Med. 2022;386:629-639. 34904798 10.1056/NEJMoa2116596

[16] Vose JM Cheah CY Clausen MR , et al. 3-year update from the EPCORE NHL-1 trial: epcoritamab leads to deep and durable responses in relapsed or refractory large B-cell lymphoma. Blood. 2024;144:4480. 10.1007/s00277-026-06798-4 PMC12868022 41634395

[17] Dickinson MJ Carlo-Stella C Morschhauser F , et al. Glofitamab for relapsed or refractory diffuse large B-cell lymphoma. N Engl J Med. 2022;387:2220-2231. [t-FL subgroup outcomes reported in supplementary data of NP30179 phase I/II study]. 36507690 10.1056/NEJMoa2206913

[18] Vitolo U Phillips EH Alonso AA , et al. PB2266: TRIAL IN PROGRESS: phase 3 trial of odronextamab plus lenalidomide versus rituximab plus lenalidomide in relapsed/refractory follicular lymphoma and marginal zone lymphoma (OLYMPIA-5). Hemasphere. 2023;7:e49668c2.

[19] Abramson JS Ku M Hertzberg M , et al. Glofitamab plus gemcitabine and oxaliplatin (GemOx) versus rituximab-GemOx for relapsed or refractory diffuse large B-cell lymphoma (STARGLO): a global phase 3, randomised, open-label trial. Lancet. 2024;404:1940-1954. 39550172 10.1016/S0140-6736(24)01774-4

[20] Kalakonda N Maerevoet M Cavallo F , et al. Selinexor in patients with relapsed or refractory diffuse large B-cell lymphoma (SADAL): a single-arm, multinational, multicentre, open-label, phase 2 trial. Lancet Haematol. 2020;7:e511-e522. 32589977 10.1016/S2352-3026(20)30120-4

[21] Stephan P Di Blasi R Roulin L , et al. TRANSCAR: real-world outcomes of CD19 CAR T-cell therapy in relapsed/refractory transformed indolent lymphomas. Blood Adv. 2025;9:4693-4704. 40311067 10.1182/bloodadvances.2025015834 PMC12466217

[22] Thiruvengadam SK Merryman R Wang Y , et al. Outcomes of CD19 CAR T in transformed indolent lymphoma compared to de novo aggressive large B-cell lymphoma. Am J Hematol. 2024;99:2178-2187. 39715004 10.1002/ajh.27548 PMC11705210

[23] Zhang J Cai D Gao R , et al. Case report: CD19 CAR T-cell therapy following autologous stem cell transplantation: a successful treatment for R/R CD20-negative transformed follicular lymphoma with TP53 mutation. Front Immunol. 2023;14:1307242. 38143763 10.3389/fimmu.2023.1307242 PMC10739420

[24] Huang Y Gong Y Liu X , et al. Case report: bispecific CD20/CD30-targeted chimeric antigen receptor T-cell therapy for non-Hodgkin's lymphoma. Front Immunol. 2025;16:1567149. 40406106 10.3389/fimmu.2025.1567149 PMC12096171

[25] Spiegel JY Patel S Muffly L , et al. CAR T cells with dual targeting of CD19 and CD22 in adult patients with recurrent or refractory B-cell malignancies: a phase 1 trial. Nat Med. 2021;27:1419-1431. 34312556 10.1038/s41591-021-01436-0 PMC8363505

[26] Nizamuddin IA Bartlett NL . Bispecific antibodies in follicular lymphoma. Haematologica. 2025;110:1472-1482. 39479864 10.3324/haematol.2024.285245 PMC12208158

[27] Ghione P Palomba ML Patel AR , et al. Comparative effectiveness of ZUMA-5 (axi-cel) vs SCHOLAR-5 external control in relapsed/refractory follicular lymphoma. Blood. 2022;140:851-860. 35679476 10.1182/blood.2021014375 PMC9412012

[28] Fukuhara N Kato K Goto H , et al. Efficacy and safety of tisagenlecleucel in adult Japanese patients with relapsed or refractory follicular lymphoma: results from the phase 2 ELARA trial. Int J Hematol. 2023;117:251-259. 36404384 10.1007/s12185-022-03481-y PMC9889457

[29] Britton K Mahat U Richardson NC , et al. FDA approval summary: lisocabtagene maraleucel for relapsed or refractory follicular lymphoma. Clin Cancer Res. 2025;31:3830-3833. 40663365 10.1158/1078-0432.CCR-25-1035 PMC12369282

[30] Bogucka-Fedorczuk A Wróbel T . Bispecific antibodies in the treatment of follicular lymphoma. Hematology in Clinical Practice. 2023;14:80-86.

[31] U.S. Food and Drug Administration . FDA approves selinexor for relapsed/refractory diffuse large B-cell lymphoma including transformed follicular lymphoma. FDA; 2020. [Regulatory approval reference; efficacy data from SADAL trial, ref 25].

[32] Pasqualucci L Khiabanian H Fangazio M , et al. Genetics of follicular lymphoma transformation. Cell Rep. 2014;6:130-140. 24388756 10.1016/j.celrep.2013.12.027 PMC4100800

[33] Kridel R Mottok A Farinha P , et al. Cell of origin of transformed follicular lymphoma. Blood. 2015;126:2118-2127. 26307535 10.1182/blood-2015-06-649905 PMC4626253

[34] Bouska A Zhang W Gong Q , et al. Combined copy number and mutation analysis identifies oncogenic pathways associated with transformation of follicular lymphoma. Leukemia. 2017;31:83-91. 27389057 10.1038/leu.2016.175 PMC5214175

[35] Schmitz R Wright GW Huang DW , et al. Genetics and pathogenesis of diffuse large B-cell lymphoma. N Engl J Med. 2018;378:1396-1407. 29641966 10.1056/NEJMoa1801445 PMC6010183

[36] Chapuy B Stewart C Dunford AJ , et al. Molecular subtypes of diffuse large B-cell lymphoma are associated with distinct pathogenic mechanisms and outcomes. Nat Med. 2018;24:679-690. 29713087 10.1038/s41591-018-0016-8 PMC6613387

[37] Lossos IS Gascoyne RD . Transformation of follicular lymphoma. Best Pract Res Clin Haematol. 2011;24:147-163. 21658615 10.1016/j.beha.2011.02.006 PMC3112479

[38] Dreval K Hilton LK Cruz M , et al. Genetic subdivisions of follicular lymphoma defined by distinct coding and noncoding mutation patterns. Blood. 2023;142:561-573. 37084389 10.1182/blood.2022018719 PMC10644066

[39] Okosun J Wolfson RL Wang J , et al. Recurrent mTORC1-activating RRAGC mutations in follicular lymphoma. Nat Genet. 2016;48:183-188. 26691987 10.1038/ng.3473 PMC4731318

[40] Brodtkorb M Lingjærde OC Huse K , et al. Whole-genome integrative analysis reveals expression signatures predicting transformation in follicular lymphoma. Blood. 2014;123:1051-1054. 24357726 10.1182/blood-2013-07-512392

[41] Federico M Caballero Barrigón MD Marcheselli L , et al. Rituximab and the risk of transformation of follicular lymphoma: a retrospective pooled analysis. Lancet Haematol. 2018;5:e359-e367. 30078408 10.1016/S2352-3026(18)30090-5

[42] Freeman CL Kridel R Moccia AA , et al. Early progression after bendamustine-rituximab is associated with high risk of transformation in advanced stage follicular lymphoma. Blood. 2019;134:761-764. 31300404 10.1182/blood.2019000258

[43] Kotlov N Bagaev A Revuelta MV , et al. Clinical and biological subtypes of B-cell lymphoma revealed by microenvironmental signatures. Cancer Discov. 2021;11(6):1468-1489. 33541860 10.1158/2159-8290.CD-20-0839 PMC8178179

[44] Sarkozy C Wu S Takata K , et al. Integrated single cell analysis reveals co-evolution of malignant B cells and tumor microenvironment in transformed follicular lymphoma. Cancer Cell. 2024;42:1003-1017.e6. 38861923 10.1016/j.ccell.2024.05.011

[45] Laurent C Trisal P Tesson B , et al. Follicular lymphoma comprises germinal center-like and memory-like molecular subtypes with prognostic significance. Blood. 2024;144(24):2503-2516. 39374535 10.1182/blood.2024024496

[46] Radtke AJ Roschewski M . The follicular lymphoma tumor microenvironment at single-cell and spatial resolution. Blood. 2024;143:1069-1079. 38194685 10.1182/blood.2023020999 PMC11103101

[47] Tilly H Morschhauser F Sehn LH , et al. Polatuzumab vedotin in previously untreated diffuse large B-cell lymphoma. N Engl J Med. 2022;386:351-363. 34904799 10.1056/NEJMoa2115304 PMC11702892

[48] Conconi A Ponzio C Lobetti-Bodoni C , et al. Incidence, risk factors and outcome of histological transformation in follicular lymphoma. Br J Haematol. 2012;157:188-196. 22348437 10.1111/j.1365-2141.2012.09054.x

[49] Horning SJ Rosenberg SA . The natural history of initially untreated low-grade non-Hodgkin's lymphomas. N Engl J Med. 1984;311:1471-1475. 6548796 10.1056/NEJM198412063112303

[50] Li Z-H Zhang M-Y Federico M , et al. Early histological transformation of follicular lymphoma to diffuse large B-cell lymphoma indicating adverse survival. Cancer. 2024;130:3321-3332. 38809573 10.1002/cncr.35378

[51] Montoto S Davies AJ Matthews J , et al. Risk and clinical implications of transformation of follicular lymphoma to diffuse large B-cell lymphoma. J Clin Oncol. 2007;25:2426-2433. 17485708 10.1200/JCO.2006.09.3260

[52] Ghesquières H Berger F Felman P , et al. Clinicopathologic characteristics and outcome of diffuse large B-cell lymphomas presenting with an associated low-grade component at diagnosis. J Clin Oncol. 2006;24:5234-5241. 17043351 10.1200/JCO.2006.07.5671

[53] Schöder H Noy A Gönen M , et al. Intensity of 18-fluorodeoxyglucose uptake in PET distinguishes between indolent and aggressive non-Hodgkin's lymphoma. J Clin Oncol. 2005;23:4643-4651. 15837966 10.1200/JCO.2005.12.072

[54] Al-Tourah AJ Gill KK Chhanabhai M , et al. Population-based analysis of incidence and outcome of transformed non-Hodgkin's lymphoma. J Clin Oncol. 2008;26:5165-5169. 18838711 10.1200/JCO.2008.16.0283

[55] Parry EM Okosun J . An updated understanding of follicular lymphoma transformation. Blood. 2025;146:1812-1823. 40373273 10.1182/blood.2024026016

[56] Goyal G Magnusson T Wang X Roose J Narkhede M Seymour E . Modern, real-world patterns of care and clinical outcomes among patients with newly diagnosed diffuse large B-cell lymphoma with or without double/triple-hit status in the United States. Haematologica. 2023;108:1190-1195. 36453108 10.3324/haematol.2022.281461 PMC10071129

[57] NCCN Clinical Practice Guidelines in Oncology . B-cell lymphomas. Version; 2025.

[58] Madsen C Pedersen MB Vase MØ , et al. Outcome determinants for transformed indolent lymphomas treated with or without autologous stem-cell transplantation. Ann Oncol. 2015;26:393-399. 25411416 10.1093/annonc/mdu537

[59] Villa D Crump M Keating A Panzarella T Feng B Kuruvilla J . Outcome of patients with transformed indolent non-Hodgkin lymphoma referred for autologous stem-cell transplantation. Ann Oncol. 2013;24:1603-1609. 23425946 10.1093/annonc/mdt029

[60] Ban-Hoefen M Vanderplas A Crosby-Thompson AL , et al. Transformed non-Hodgkin lymphoma in the rituximab era: analysis of the NCCN outcomes database. Br J Haematol. 2013;163:487-495. 24111533 10.1111/bjh.12570

[61] Gisselbrecht C Glass B Mounier N , et al. Salvage regimens with autologous transplantation for relapsed large B-cell lymphoma in the rituximab era. J Clin Oncol. 2010;28:4184-4190. 20660832 10.1200/JCO.2010.28.1618 PMC3664033

[62] Neelapu SS Locke FL Bartlett NL , et al. Axicabtagene ciloleucel CAR T-cell therapy in refractory large B-cell lymphoma. N Engl J Med. 2017;377:2531-2544. 29226797 10.1056/NEJMoa1707447 PMC5882485

[63] Thieblemont C Phillips T Ghesquieres H , et al. Epcoritamab, a novel, subcutaneous CD3xCD20 bispecific T-cell-engaging antibody, in relapsed or refractory large B-cell lymphoma: dose expansion in a phase I/II trial. J Clin Oncol. 2023;41:2238-2247. 36548927 10.1200/JCO.22.01725 PMC10115554

[64] Noy A Schöder H Gönen M , et al. The majority of transformed lymphomas have high standardized uptake values (SUVs) on PET similar to diffuse large B-cell lymphoma (DLBCL). Ann Oncol. 2009;20:508-512. 19139176 10.1093/annonc/mdn657 PMC4542578

[65] NCCN Clinical Practice Guidelines in Oncology. B-cell lymphomas. Version 2025. National Comprehensive Cancer Network.

