# Supplementary material for: Beyond Chemoimmunotherapy: Emerging Cellular and Targeted Therapies in Transformed Follicular Lymphoma: A Scoping Review
Source: Cancer Control. 2026 Jun 9;33:10732748261454479. doi: 10.1177/10732748261454479 (PMC13254433; doi:10.1177/10732748261454479)
Supplement: Supplemental Material - Beyond Chemoimmunotherapy: Emerging Cellular and Targeted Therapies in Transformed Follicular Lymphoma: A Scoping Review [file sj-pdf-1-ccx-10.1177_10732748261454479.pdf]

Preferred Reporting Items for Systematic Reviews and Meta-Analyses Extension for Scoping Reviews (PRISMA-ScR) — Completed Checklist

Manuscript: Beyond Chemoimmunotherapy: Emerging Cellular and Targeted Therapies in Transformed Follicular Lymphoma: A Scoping Review

Al-Mashdali AF, Ghasoub R, Hwafdeh S, Yassin MA | NCCCR, Hamad Medical Corporation, Doha, Qatar | Correspondence: AAlmashdali@hamad.qa

SECTION	ITEM	PRISMA-ScR CHECKLIST ITEM	REPORTED ON PAGE # / LOCATION
**TITLE**			
Title	**1**	Identify the report as a scoping review.	Page 1 — Title: "Beyond Chemoimmunotherapy: Emerging Cellular and Targeted Therapies in Transformed Follicular Lymphoma: A Scoping Review." The term "Scoping Review" is explicitly stated in the title.
**ABSTRACT**			
Structured summary	**2**	Provide a structured summary that includes (as applicable): background, objectives, eligibility criteria, sources of evidence, charting methods, results, and conclusions that relate to the review questions and objectives.	Pages 1–2 — Structured abstract with labelled sections: Introduction (background and objective), Methods (eligibility criteria, databases searched, eligible designs), Results (17 studies, 663 patients, outcomes by therapy class), and Conclusions (stepwise treatment framework, research priorities).
**INTRODUCTION**			
Rationale	**3**	Describe the rationale for the review in the context of what is already known. Explain why the review questions/objectives lend themselves to a scoping review approach.	Pages 2–3 (Introduction) — The rationale describes the limited prospective evidence for t-FL management, the reliance on subset analyses, and the exclusion of transformed histology from pivotal trials. A scoping review approach is appropriate because the objective is to map the breadth of available evidence across heterogeneous study designs rather than generate a pooled estimate, consistent with PRISMA-ScR guidance.
Objectives	**4**	Provide an explicit statement of the questions and objectives being addressed with reference to their key elements (e.g., population or participants, concepts, and context) or other relevant key elements used to conceptualize the review questions and/or objectives.	Pages 3–4 (Methods — Review questions and PCC framework) — Objectives are structured using the PCC framework: Population (adults ≥18 yr with t-FL), Concept (diagnostic strategies and therapeutic approaches — chemoimmunotherapy, ASCT, CAR-T, bispecific antibodies, selinexor — with extractable efficacy and safety outcomes), and Context (all care settings and geographies, peer-reviewed publications and conference abstracts, January 2000–May 2025).

SECTION	ITEM	PRISMA-ScR CHECKLIST ITEM	REPORTED ON PAGE # / LOCATION
**METHODS**			
Protocol and registration	**5**	Indicate whether a review protocol exists; state if and where it can be accessed (e.g., a Web address); and if available, provide registration information, including the registration number.	Page 3 (Methods — Protocol and registration) — A formal protocol was developed a priori and is available from the corresponding author (AAlmashdali@hamad.qa) on request. The review was not pre-registered; however, all eligibility criteria, search strategy elements, and data-charting variables were defined before screening commenced.
Eligibility criteria	**6**	Specify characteristics of the sources of evidence used as eligibility criteria (e.g., years considered, language, and publication status), and provide a rationale.	Pages 4–5 (Methods — Eligibility criteria) — Eligible designs included interventional trials, registries, and phase II/III randomised studies. Studies required at least one extractable efficacy or safety endpoint and a reportable t-FL subgroup. Reports from January 2000 through May 2025 in any language were considered. Exclusions (case reports, <10 patients without subgroup data, paediatric-only, purely diagnostic studies) are specified with rationale.
Information sources	**7**	Describe all information sources in the search (e.g., databases with dates of coverage and contact with authors to identify additional sources), as well as the date the most recent search was executed.	Pages 4–5 (Methods — Information sources and search strategy) — Three electronic databases were searched: PubMed/MEDLINE, EMBASE, and the Cochrane Library, from January 2000 through May 2025. Conference proceedings (ASH and EHA, 2020–2025) were manually screened. Reference lists of included articles and recent narrative reviews were hand-searched. The most recent search was executed on 31 May 2025.
Search	**8**	Present the full electronic search strategy for at least 1 database, including any limits used, such that it could be repeated.	Page 5 (Methods — Information sources and search strategy) and Supplementary Appendix S1 — The search strategy combining MeSH/Emtree controlled vocabulary and free-text terms under three concepts (population: "transformed follicular lymphoma" OR "histologic transformation"; interventions: CAR-T products, bispecific antibodies, selinexor, ASCT; outcomes: efficacy and safety terms) joined with Boolean operators is described in the text. Full database-specific strategies are provided in Supplementary Appendix S1.
Selection of sources of evidence	**9**	State the process for selecting sources of evidence (i.e., screening and eligibility) included in the scoping review.	Page 5–6 (Methods and Results — Selection of sources of evidence) — All retrieved citations were imported into EndNote 21 and de-duplicated. Two reviewers (A.F.A. and R.G.) independently screened titles/abstracts; full texts

SECTION	ITEM	PRISMA-ScR CHECKLIST ITEM	REPORTED ON PAGE # / LOCATION
			of potentially eligible records were assessed against the eligibility criteria. Reasons for full-text exclusion were recorded and are summarised in the PRISMA-ScR flow diagram (Figure 1).
Data charting process	**10**	Describe the methods of charting data from the included sources of evidence (e.g., calibrated forms or forms that have been tested by the team before their use, and whether data charting was done independently or in duplicate) and any processes for obtaining and confirming data from investigators.	Page 5 (Methods — Information sources and search strategy) — Data were charted using a standardised extraction form developed a priori. Charting was performed by A.F.A. and R.G. independently, with discrepancies resolved by discussion and adjudication by M.A.Y. where needed. For studies with mixed populations, only t-FL subgroup outcomes were extracted when separately reported. No contact with authors was required.
Data items	**11**	List and define all variables for which data were sought and any assumptions and simplifications made.	Pages 5–6 (Methods — Information sources and search strategy) — Variables charted included: bibliographic information (first author, year, country); study design and setting; sample size and proportion of t-FL patients; method of t-FL ascertainment (biopsy-confirmed vs. clinical); intervention and treatment line; efficacy endpoints (ORR, CR, DOR, PFS, EFS, OS); and safety outcomes (CRS grade, ICANS, grade ≥3 AEs, treatment-related mortality). Definitions are provided in Table 1 (PCC Framework).
Critical appraisal of individual sources	**12**	If done, provide a rationale for conducting a critical appraisal of included sources of evidence; describe the methods used and how this information was used in any data synthesis (if appropriate).	Page 6 and Limitations (page 17) — Consistent with PRISMA-ScR guidance and the descriptive intent of this scoping review (mapping breadth of evidence rather than generating pooled estimates), no formal risk-of-bias assessment was performed. Limitations of the underlying evidence — including selection bias, small t-FL subgroups, heterogeneous transformation definitions, and reliance on subgroup analyses and conference abstracts — are explicitly discussed in the Limitations section.
Synthesis of results	**13**	Describe the methods of handling and summarising the data that were charted.	Pages 5–6 (Methods) — Findings were synthesised narratively. No quantitative meta-analysis was performed owing to marked clinical and methodological heterogeneity across included studies. Charted results are summarised in evidence tables (Tables 2–5), a practical-positioning matrix (Table 6), and a proposed management algorithm (Figure 2). The narrative synthesis groups evidence by therapeutic class (CAR-T, bispecific antibodies, selinexor) and contextualises within the broader

SECTION	ITEM	PRISMA-ScR CHECKLIST ITEM	REPORTED ON PAGE # / LOCATION
			treatment landscape.
**RESULTS**			
Selection of sources of evidence	**14**	Give numbers of sources of evidence screened, assessed for eligibility, and included in the review, with reasons for exclusions at each stage, ideally using a flow diagram.	Pages 6–7 (Results — Selection of sources of evidence) and Figure 1 (PRISMA-ScR flow diagram) — From 680 database records and 45 conference/reference records, 276 duplicates were removed. Of 449 records screened, 89 full texts were assessed; 17 studies were included. Reasons for exclusion at full-text stage: no extractable t-FL subgroup data (n=45), fewer than 10 t-FL patients (n=14), case reports/editorials without primary data (n=10), and paediatric/language/pre-2000 (n=3).
Characteristics of sources of evidence	**15**	For each source of evidence, present characteristics for which data were charted and provide the citations.	Pages 7–10 (Results) and Tables 2–5 — Characteristics of all 17 included sources are presented across four evidence tables by therapeutic class: CAR-T interventional trials (Table 2; 7 studies), bispecific antibody trials (Table 3; 4 studies), selinexor trial (Table 4; 1 study), and real-world/case/preclinical evidence (Table 5; 5 reports). Each table presents study design, population, t-FL case numbers, intervention, efficacy, and key outcomes with full citations.
Critical appraisal within sources of evidence	**16**	If done, present data on critical appraisal of included sources of evidence (see item 12).	*Not formally performed — see Item 12. Consistent with scoping review methodology and PRISMA-ScR guidance, formal risk-of-bias assessment was not undertaken. Key methodological limitations of individual studies (e.g., single-arm design, small t-FL subgroups, post-hoc reporting, abstract-level data) are noted within the relevant evidence tables and discussed in the Limitations section (page 17).*
Results of individual sources of evidence	**17**	For each included source of evidence, present the relevant data that were charted that relate to the review questions and objectives.	Pages 7–11 (Results) and Tables 2–5 — For each included source, the following are reported: t-FL sample size, ORR, CR rate, DOR, PFS (where available), key safety outcomes (CRS, ICANS, grade ≥3 AEs), and contextual notes (e.g., CD19-negative relapse, subgroup limitations). Data from randomised trials (ZUMA-7, TRANSFORM, BELINDA) are presented with comparator arm outcomes. Real-world data are distinguished from trial data throughout.
Synthesis of results	**18**	Summarise and/or present the charting results as they relate to the review questions and	Pages 11–15 (Summary of Findings) — Charted

SECTION	ITEM	PRISMA-ScR CHECKLIST ITEM	REPORTED ON PAGE # / LOCATION
		objectives.	results are synthesised narratively across five subsections: biology and pathogenesis, epidemiology and clinical presentation, risk factors and diagnosis, current and emerging treatment strategies, and practical positioning. An evidence-informed management algorithm (Figure 2) and a practical-positioning matrix (Table 6) are provided to address the objective of proposing a clinical decision framework for t-FL management.
**DISCUSSION**			
Summary of evidence	**19**	Summarise the main results (including an overview of concepts, themes, and types of evidence available), link to the review questions and objectives, and consider the relevance to key groups.	Pages 11–16 (Summary of Findings and Conclusion) — The main results are summarised by therapeutic class, linking directly to the PCC framework objectives. Key themes include: CAR-T as the preferred option at relapse (strongest evidence); bispecific antibodies as off-the-shelf alternatives; selinexor for later-line or frail/ineligible patients; persistent exclusion of t-FL from pivotal trials; and the need for prospective t-FL-specific studies. Relevance to haematologists, oncologists, and clinical trialists is addressed throughout.
Limitations	**20**	Discuss the limitations of the scoping review process.	Pages 16–17 (Limitations) — Process limitations include: no formal risk-of-bias assessment (consistent with scoping review design); heterogeneity in transformation definitions and diagnostic methods across included studies; reliance on subgroup analyses and conference abstracts with immature follow-up; absence of t-FL-specific randomised evidence for most questions; possible population overlap between real-world cohorts; and no meta-analytic synthesis. The descriptive nature of the review and the absence of pre-registration are acknowledged.
Conclusions	**21**	Provide a general interpretation of the results with respect to the review questions and objectives, as well as potential implications and/or next steps.	Page 17 (Conclusion) — A coherent stepwise treatment framework is proposed: biopsy confirmation at transformation; DLBCL-like induction tailored to biology and anthracycline exposure; ASCT for fit, chemosensitive responders; CAR-T at first relapse for eligible patients; bispecific antibodies after CAR-T failure or when CAR-T is not feasible; selinexor for later-line or oral-therapy settings. Next steps identified include prospective t-FL-inclusive trials and biomarker-guided sequencing studies.

SECTION	ITEM	PRISMA-ScR CHECKLIST ITEM	REPORTED ON PAGE # / LOCATION
**FUNDING**			
Funding	**22**	Describe sources of funding for the included sources of evidence, as well as sources of funding for the scoping review. Describe the role of the funders of the scoping review.	Page 17 (Statements and Declarations — Funding) — This scoping review received no specific grant from public, commercial, or not-for-profit funding agencies. Open-access publication fees will be covered by Qatar National Library upon acceptance. Funding sources of individual included studies are not systematically reported, as this is a scoping review; study-level funding can be found in the original publications cited in References [7–29]. Funders had no role in study design, data extraction, interpretation, or the decision to submit.

Abbreviations: ASCT, autologous stem-cell transplant; CAR-T, chimeric antigen receptor T-cell therapy; CR, complete response; CRS, cytokine release syndrome; DOR, duration of response; EFS, event-free survival; ICANS, immune effector cell-associated neurotoxicity syndrome; JBI, Joanna Briggs Institute; NCCCR, National Center for Cancer Care and Research; ORR, overall response rate; OS, overall survival; PCC, Population–Concept–Context; PFS, progression-free survival; PRISMA-ScR, Preferred Reporting Items for Systematic Reviews and Meta-Analyses extension for Scoping Reviews; t-FL, transformed follicular lymphoma.

Reference: *Tricco AC, Lillie E, Zarin W, et al. PRISMA Extension for Scoping Reviews (PRISMA-ScR): Checklist and Explanation. Ann Intern Med. 2018;169:467–473. doi:10.7326/M18-0850.*
